# Association Between the Triglyceride-Glucose Index and Incident Cardiovascular Disease in Non-diabetic Adults: A Prospective Cohort Study

**DOI:** 10.7759/cureus.115208

**Published:** 2026-08-26

**Authors:** Muhammad Ammad Faisal, Shrikar Iragavarapu, Kamran Khan, Nereen Awan, Sana Tariq, Asad N Bajwa, Saad Bajwa, FNU Mahparah, Shewani Ahuja, Shahid Hussain, Agha Saffi Ullah Khan

**Affiliations:** 1 Cardiovascular Medicine, Faisalabad Institute of Cardiology, Faisalabad, PAK; 2 Internal Medicine, University of Alabama at Birmingham, Birmingham, USA; 3 Internal Medicine, Poonch Medical College, Rawalakot, PAK; 4 Obstetrics and Gynecology, Combined Military Hospital Rawalpindi, Rawalpindi, PAK; 5 Acute Medicine and Critical Care, Rawalpindi Medical University, Rawalpindi, PAK; 6 Internal Medicine, D. G. Khan Medical College, Dera Ghazi Khan, PAK; 7 Internal Medicine, Rawalpindi Medical University, Rawalpindi, PAK; 8 Internal Medicine, Punjab Medical College, Faisalabad, PAK; 9 Internal Medicine, People’s University of Medical and Health Sciences for Women, Nawabshah, PAK; 10 Family Medicine, Jinnah Sindh Medical University, Karachi, PAK; 11 Internal Medicine, Avicenna Medical College, Lahore, PAK

**Keywords:** cardiovascular disease, cohort study, insulin resistance, non-diabetic adults, triglyceride-glucose index

## Abstract

Background: Cardiovascular disease (CVD) often develops silently; therefore, early markers of cardiovascular risk should be identified before the onset of overt metabolic disorders. The study evaluated the association between the triglyceride-glucose (TyG) index and incident CVD among non-diabetic adults without established CVD.

Methods: A total of 553 non-diabetic adults without established CVD were recruited using non-probability convenience sampling from tertiary care centres and followed for 12-29 months. We collected demographic, clinical, and laboratory parameters, and computed the TyG index from fasting triglyceride and glucose levels. A total of 125 (22.6%) participants developed incident CVD during follow-up.

Results: We observed a clear dose-response relationship, with 10/139 (7.2%) participants in the lowest TyG quartile and 54/138 (39.1%) participants in the highest TyG quartile developing incident CVD. After adjustment for age, gender, BMI, smoking status, hypertension, physical activity, and family history of CVD, the TyG index remained strongly associated with incident CVD (adjusted HR = 4.597, p < 0.001), while only hypertension remained statistically significant. The receiver operating characteristic curve analysis demonstrated moderate discriminative ability for incident CVD (area under the curve (AUC): 0.694).

Conclusion: The results suggest that the TyG index may serve as a complementary, low-cost marker for cardiovascular risk assessment in non-diabetic individuals; however, its moderate discriminative ability and lack of demonstrated incremental prognostic value warrant further validation.

## Introduction

Cardiovascular disease (CVD) remains one of the leading causes of morbidity and mortality worldwide and can develop without symptoms [1,2]. Traditional risk assessment models are based on past factors such as hypertension, dyslipidemia, and diabetes. However, they are unable to identify people with underlying metabolic disorders, as these people have not yet reached diagnostic thresholds [3,4]. Furthermore, subclinical metabolic dysfunction (as measured by insulin resistance and composite cardiometabolic abnormalities) may contribute significantly to the progression to subsequent cardiovascular events at the early or preclinical stage [5,6].

Insulin resistance is one mechanism linked to early metabolic imbalance and cardiovascular pathology [7]. However, the development of CVD is multifactorial. Also, it involves oxidative stress, endothelial dysfunction, chronic low-grade inflammation, adipokine dysregulation, and hepatic insulin resistance, all of which contribute to atherosclerosis and vascular injury [8,9]. These mechanisms promote atherogenic lipid profiles and vascular injury even in the absence of diabetes [9]. Because directly measuring insulin resistance is challenging, the triglyceride-glucose (TyG) index, based on fasting triglyceride and glucose values, is recognized as a convenient and inexpensive surrogate endpoint [10,11]. The TyG index is a simple, inexpensive, and clinically useful surrogate marker for identifying individuals with early metabolic dysfunction [12].

Although previous large prospective cohort studies, including the PURE study, have demonstrated an association between the TyG index and cardiovascular outcomes, and hospital-based cohorts have also reported its prognostic value, prospective evidence from Pakistani non-diabetic adults remains limited [13,14]. Therefore, this study aimed to evaluate the association between the TyG index and incident CVD in non-diabetic adults and to assess its potential utility as a clinically feasible marker for early cardiovascular risk assessment.

Objectives

The primary objective of this study was to determine whether baseline TyG index was independently associated with incident composite CVD among non-diabetic adults after adjustment for traditional cardiovascular risk factors. Secondary objectives were to assess the dose-response relationship between TyG index and incident CVD and to evaluate its discriminatory performance for incident CVD.

## Materials and methods

Study design and setting

A prospective cohort study was conducted from 25 April 2024 to 10 March 2026 in tertiary care hospitals and diagnostic centers in Rawalpindi and Islamabad to assess the performance of the TyG index in predicting the incidence of CVDs. Potential participants were approached during routine visits to the participating healthcare facilities and were screened against the predefined eligibility criteria before enrollment. Eligible participants who agreed to participate were enrolled after providing written informed consent. All participants were recruited at baseline and followed longitudinally, with follow-up assessments conducted at six- to 12-month intervals to identify incident cardiovascular events. The follow-up duration ranged from 12 to 29 months, with a mean follow-up duration of 23.69 ± 7.33 months. No participants were lost to follow-up during the study period. The relationship between the baseline TyG and progression to CVD could be evaluated over time using this design.

Study population and sampling

The participants were adult patients who visited the selected healthcare facilities for general check-ups or for symptoms unrelated to CVD. Non-probability convenience sampling was used to select the participants. As participants were recruited from tertiary care hospitals, the study population may have had a relatively higher baseline cardiovascular risk than the general community, which should be considered when interpreting the findings. Participants were adults aged ≥18 years who were not diagnosed with diabetes at enrollment. To determine incident cases of CV disease during follow-up, individuals with a known history of diabetes mellitus (DM) or established CV disease were also excluded. Other exclusions included severe comorbid conditions that might affect metabolic parameters or limit follow-up, as well as inadequate baseline data. Before inclusion, all participants who met the inclusion criteria were informed of the study's purpose and procedures, and informed consent was obtained from all participants.

Sample size

A total of 553 participants were included in the study. The final dataset comprised 125 incident cardiovascular events, providing an adequate number of outcome events for the planned multivariable Cox proportional hazards regression analysis. The adjusted model included 10 parameters, yielding approximately 12.5 events per parameter, which exceeds the commonly recommended minimum events-per-variable threshold for regression modelling [15].

Eligibility criteria

Eligible participants were adults aged 30-75 years without established CVD or previously diagnosed DM at baseline (fasting plasma glucose <126 mg/dL) who provided written informed consent. Individuals with chronic kidney disease, chronic liver disease, active malignancy, pregnancy, or those receiving systemic corticosteroids or lipid-lowering therapy were excluded. Participants with incomplete baseline clinical or laboratory data or insufficient follow-up information were also excluded.

Data collection

Data were collected using structured questionnaires, clinical examinations, and laboratory investigations. Demographic aspects (age, gender, marital status, occupation, and socioeconomic status) and lifestyle risk factors (smoking status, physical activity, and family history of CVD) were captured in the questionnaire (see Appendices).

Baseline clinical measurements were obtained directly, including blood pressure (BP) via a standard sphygmomanometer and hypertension status. Anthropometric data, including weight and height, were also collected using calibrated anthropometric instruments, and BMI was calculated.

Laboratory parameters were collected after an 8-12-hour overnight fast and included fasting blood glucose and triglyceride levels, from which the TyG index was calculated. Participants were instructed to maintain an overnight fast of 8-12 hours before blood sample collection, and fasting status was confirmed verbally by the attending healthcare staff before venipuncture.

Data collection procedure

Baseline data collection comprised three areas: questionnaire, clinical, and laboratory assessments. All participants were carefully screened upon entry, and baseline variables were recorded. BP and anthropometric measurements were obtained during the clinical examination; laboratory measurements were obtained after 8-12 hours of fasting. Fasting blood glucose and fasting triglycerides were included in the laboratory data, and the TyG index was subsequently calculated using the standard formula [16]: $\mathrm{TyG\ Index}=\ln\left(\frac{\mathrm{Fasting\ Triglycerides\ (mg/dL)}\times\mathrm{Fasting\ Blood\ Glucose\ (mg/dL)}}{2}\right)$.

Incident cardiovascular events were identified by following participants every 12-month interval after the baseline assessment through telephone interviews and hospital record reviews. The composite primary clinical outcome was myocardial infarction, stroke, angina, and documented coronary artery disease. Incident CVD was defined as a newly documented diagnosis of myocardial infarction, stroke, angina, or coronary artery disease occurring after baseline. Events were confirmed by review of hospital medical records, discharge summaries, or relevant diagnostic documentation; telephone-reported events were considered confirmed only when supporting medical documentation was available. The composite endpoint represented the broad spectrum of clinically relevant cardiovascular manifestations. It was verified in medical records or discharge summaries, where available. Participants who remained free of CVD at the end of follow-up were censored at their last completed follow-up assessment. As no participants were lost to follow-up, no censoring due to loss to follow-up was required.

Data analysis

IBM SPSS Statistics for Windows, Version 26 (Released 2018; IBM Corp., Armonk, New York, United States) was used to record, clean, and analyze data. All continuous variables were assessed for skewness to assess normality and reported as mean, standard deviation (SD), median, and range. In contrast, categorical variables were described as frequencies and percentages. Baseline demographic and clinical data were summarized using descriptive statistics. There was no missing data for variables used in the final analysis. Spearman's rank correlation was used to assess the correlation between TyG and continuous variables. Spearman's rank correlation was selected because it does not assume a normal distribution and provides a robust measure of monotonic associations between continuous variables. Participants were divided into quartiles based on TyG index values, and differences in CVD incidence across quartiles were compared using the chi-square (χ²) test to explore dose-response relationships. To estimate unadjusted and adjusted hazard ratios (HRs) with 95% confidence intervals (CIs), time-to-event analysis was performed using Cox proportional hazards regression. Covariates included in the adjusted Cox regression model were selected a priori based on established cardiovascular risk factors and their potential clinical relevance to the TyG-CVD association. The model included age, gender, BMI, smoking status, hypertension, physical activity, and family history of CVD. Receiver operating characteristic (ROC) curve analysis was performed to evaluate the discriminative ability of the TyG index for incident CVD, with the area under the curve (AUC) used to quantify its discrimination. Incidence rates were calculated as the number of incident CVD events divided by the total accumulated person-time at risk, with follow-up time converted from months to years, and were expressed per 1,000 person-years. All analyses were considered statistically significant if the p-value was below 0.05.

Ethical approval

The study was approved by the institutional review board of Avicenna Medical College (7B01-IEB-AMC-2024) at each participating center before the start of the study. Ethical guidelines on human research were followed in all the procedures. All participants were informed of the study and signed informed consent forms before enrolling. The participants' data were carefully protected to ensure confidentiality and anonymity, and were used only for research purposes.

## Results

The demographic and clinical characteristics of the study population (N = 553) are summarized in Table 1. The sample comprised 283 males (51.2%) and 270 females (48.8%). A majority of participants were non-smokers (352, 63.7%), while 201 (36.3%) were smokers. Hypertension was present in 189 (34.2%) participants, whereas 364 (65.8%) did not have hypertension. Regarding physical activity, 327 (59.1%) participants were sedentary, while 226 (40.9%) were physically active. A family history of CVD was reported by 184 (33.3%) participants, whereas 369 (66.7%) had no family history of CVD. Participant distribution across TyG index quartiles was fairly even, with 139 (25.1%) participants in the lowest quartile (Q1) and 138 (25.0%) in each of the remaining quartiles (Q2, Q3, and Q4). During follow-up, 125 participants (22.6%) experienced incident CVD, while 428 (77.4%) remained free of CVD.

**Table 1:** Baseline Demographic and Clinical Characteristics of Study Participants (N = 553) Data are presented as frequency (n) and valid percentage (%). No missing values were observed for any variable. CVD: cardiovascular disease; TyG: triglyceride-glucose index

Variable	Overall (N = 553), n (%)	No incident CVD (n = 428), n (%)	Incident CVD (n = 125), n (%)	p-value
Gender				
Male	283 (51.2)	210 (49.1)	73 (58.4)	0.083
Female	270 (48.8)	218 (50.9)	52 (41.6)	
Smoking status				
Non-smoker	352 (63.7)	287 (67.1)	65 (52.0)	0.003
Smoker	201 (36.3)	141 (32.9)	60 (48.0)	
Hypertension				
No	364 (65.8)	308 (72.0)	56 (44.8)	<0.001
Yes	189 (34.2)	120 (28.0)	69 (55.2)	
Physical activity				
Sedentary	327 (59.1)	239 (55.8)	88 (70.4)	0.005
Active	226 (40.9)	189 (44.2)	37 (29.6)	
Family history of CVD				
No	369 (66.7)	302 (70.6)	67 (53.6)	<0.001
Yes	184 (33.3)	126 (29.4)	58 (46.4)	
TyG index quartile				
Q1 - Lowest	139 (25.1)	121 (28.3)	18 (14.4)	<0.001
Q2	138 (25.0)	113 (26.4)	25 (20.0)	
Q3	138 (25.0)	105 (24.5)	33 (26.4)	
Q4 - Highest	138 (25.0)	89 (20.8)	49 (39.2)	

Table 2 presents descriptive statistics for continuous variables among participants in the study (N = 553). The mean age was 53.01 years (SD = 10.25), with a median of 53.00 and a range of 30 to 75 years, and showed a rather symmetric distribution (skewness = -0.003). The mean weight was 74.93 kg (SD = 10.51), and the mean height was 1.664 m (SD = 0.078), resulting in a mean BMI of 27.02 kg/m² (SD = 2.99), indicating an overall overweight population. The mean systolic BP (SBP) was 139.29 mmHg (SD = 16.72), and the mean diastolic BP (DBP) was 85.69 mmHg (SD = 5.91) during BP measurement. Biochemical parameters showed a mean fasting glucose of 94.53 mg/dL (SD = 8.68), a mean triglyceride of 206.79 mg/dL (SD = 72.69), and a mean TyG index of 9.120 (SD = 0.384). The follow-up duration ranged from 12 to 29 months (mean: 23.69 ± 7.33 months); the mean event time was 22.20 months (SD = 7.12), and no participants were lost to follow-up. All variables demonstrated acceptable distributional symmetry, with skewness values ranging from -0.076 to 0.463. Furthermore, all participants had fasting plasma glucose levels below 126 mg/dL, confirming the exclusion of individuals with diabetes.

**Table 2:** Descriptive Statistics of Continuous Variables (N = 553) Skewness values between -2.0 and +2.0 indicate acceptable distributional symmetry. All fasting glucose values were <126 mg/dL, confirming the exclusion of participants with diabetes. M: mean; SD: standard deviation; Min: minimum; Max: maximum; BMI: body mass index; BP: blood pressure; TyG: triglyceride-glucose index = ln(triglycerides × fasting glucose/2)

Variable	M	SD	Median	Min	Max	Skewness
Age (years)	53.01	10.25	53.00	30	75	-0.003
Weight (kg)	74.93	10.51	74.00	50.2	100.0	0.237
Height (m)	1.664	0.078	1.670	1.50	1.85	-0.017
BMI (kg/m²)	27.02	2.99	26.96	21.98	32.01	0.006
Systolic BP (mmHg)	139.29	16.72	139.00	110	170	0.010
Diastolic BP (mmHg)	85.69	5.91	85.00	70	99	0.103
Fasting glucose (mg/dL)	94.53	8.68	94.00	80	122	0.201
Triglycerides (mg/dL)	206.79	72.69	197.00	100	350	0.463
TyG index	9.120	0.384	9.133	8.294	9.901	-0.076
Follow-up (months)	23.69	7.33	23.00	12	29	0.080
Event time (months)	22.20	7.12	21.00	12	29	0.398

Table 3 presents the Spearman correlation matrix evaluating the association between the TyG index and continuous variables (N = 553). The TyG index showed a weak-to-moderate positive correlation with BMI (ρ = 0.263, p < 0.001) and body weight (ρ = 0.184, p < 0.001). Age demonstrated only a weak positive correlation with the TyG index (ρ = 0.107, p < 0.05), indicating a negligible effect size despite statistical significance. There were weak, non-significant correlations between the TyG index and SBP (ρ = 0.079) and DBP (ρ = 0.044), while its correlation with height was weak and negative (ρ = -0.021). BMI showed a strong positive correlation with body weight (ρ = 0.747, p < 0.001), whereas height was moderately correlated with body weight (ρ = 0.595, p < 0.001). Age demonstrated weak positive correlations with SBP (ρ = 0.161, p < 0.001) and DBP (ρ = 0.154, p < 0.001), while showing weak negative correlations with BMI (ρ = -0.120, p < 0.001) and body weight (ρ = -0.101, p < 0.05). Overall, the TyG index demonstrated weak-to-moderate correlations with anthropometric measures and weak or non-significant correlations with BP variables.

**Table 3:** Spearman Correlation Matrix of TyG Index and Continuous Variables (N = 553) Correlation coefficients are Spearman's rho (ρ). The upper triangle is omitted for clarity. Values with asterisks are statistically significant. *p < 0.05. **p < 0.001. TyG: triglyceride-glucose index; BMI: body mass index; SBP: systolic blood pressure; DBP: diastolic blood pressure

Variable	TyG index	BMI	Body weight	Age	SBP	DBP	Height
1. TyG index	-	-	-	-	-	-	-
2. BMI	0.263**	-	-	-	-	-	-
3. Body weight	0.184**	0.747**	-	-	-	-	-
4. Age	0.107*	-0.120**	-0.101*	-	-	-	-
5. SBP	0.079	0.156**	0.060	0.161**	-	-	-
6. DBP	0.044	0.054	0.000	0.154**	0.558**	-	-
7. Height	-0.021	-0.052	0.595**	-0.013	-0.081	-0.076	-

Table 4 presents the incidence of CVD across TyG index quartiles among participants (N = 553). In the lowest quartile (Q1), 10 participants (7.2%) experienced CVD events, while 129 (92.8%) remained event-free. In Q2, CVD occurred in 28 participants (20.3%), compared with 110 (79.7%) without events. Similarly, 33 participants (23.9%) in Q3 developed CVD, while 105 (76.1%) remained event-free. The highest quartile (Q4) had the greatest burden, with 54 participants (39.1%) experiencing CVD events and 84 (60.9%) remaining event-free. Overall, 125 participants (22.6%) developed CVD, while 428 (77.4%) did not. The mean TyG index increased progressively across quartiles, from 8.616 (SD = 0.150) in Q1 to 9.615 (SD = 0.131) in Q4. CVD incidence differed significantly across TyG quartiles (χ² = 40.969, p < 0.001). Furthermore, the Cochran-Armitage trend test demonstrated a significant linear trend across increasing TyG quartiles (χ²trend = 31.84, p < 0.001), with CVD incidence progressively increasing from Q1 to Q4, supporting a dose-response relationship between higher TyG levels and incident CVD.

**Table 4:** CVD Incidence Across TyG Index Quartiles (N = 553) Note: RR: relative risk of incident CVD, with Q1 as the reference quartile. Absolute risk difference represents the difference in cumulative CVD risk relative to Q1, expressed in percentage points. Incidence rates are presented as incident CVD events per 1000 person-years of follow-up. The Cochran-Armitage trend test (χ²trend = 31.84, p < 0.001) was applied to evaluate the ordered dose-response relationship across TyG quartiles. The Pearson chi-square statistic and corresponding p-value represent the overall association between TyG quartile and incident CVD across all four quartiles and do not refer specifically to the Total row. CVD: cardiovascular disease; TyG: triglyceride-glucose index

TyG quartile	n	TyG, M (SD)	CVD events, n (%)	No CVD, n (%)	RR (95% CI)	Absolute risk difference, % points	Cochran-Armitage trend test (χ²trend)	p	Incidence rate (per 1000 person-years)
Q1 - Lowest	139	8.616 (0.150)	10 (7.2)	129 (92.8)	1.00 (Reference)	0.0 (Reference)	-	-	26.5
Q2	138	8.989 (0.080)	28 (20.3)	110 (79.7)	2.82 (1.43-5.58)	+13.1	-	-	88.4
Q3	138	9.265 (0.075)	33 (23.9)	105 (76.1)	3.32 (1.71-6.48)	+16.7	-	-	102.7
Q4 - Highest	138	9.615 (0.131)	54 (39.1)	84 (60.9)	5.44 (2.89-10.24)	+31.9	-	-	166.8
Total	553	9.120 (0.384)	125 (22.6)	428 (77.4)	-	-	31.84	<0.001	114.5

**Figure 1:**
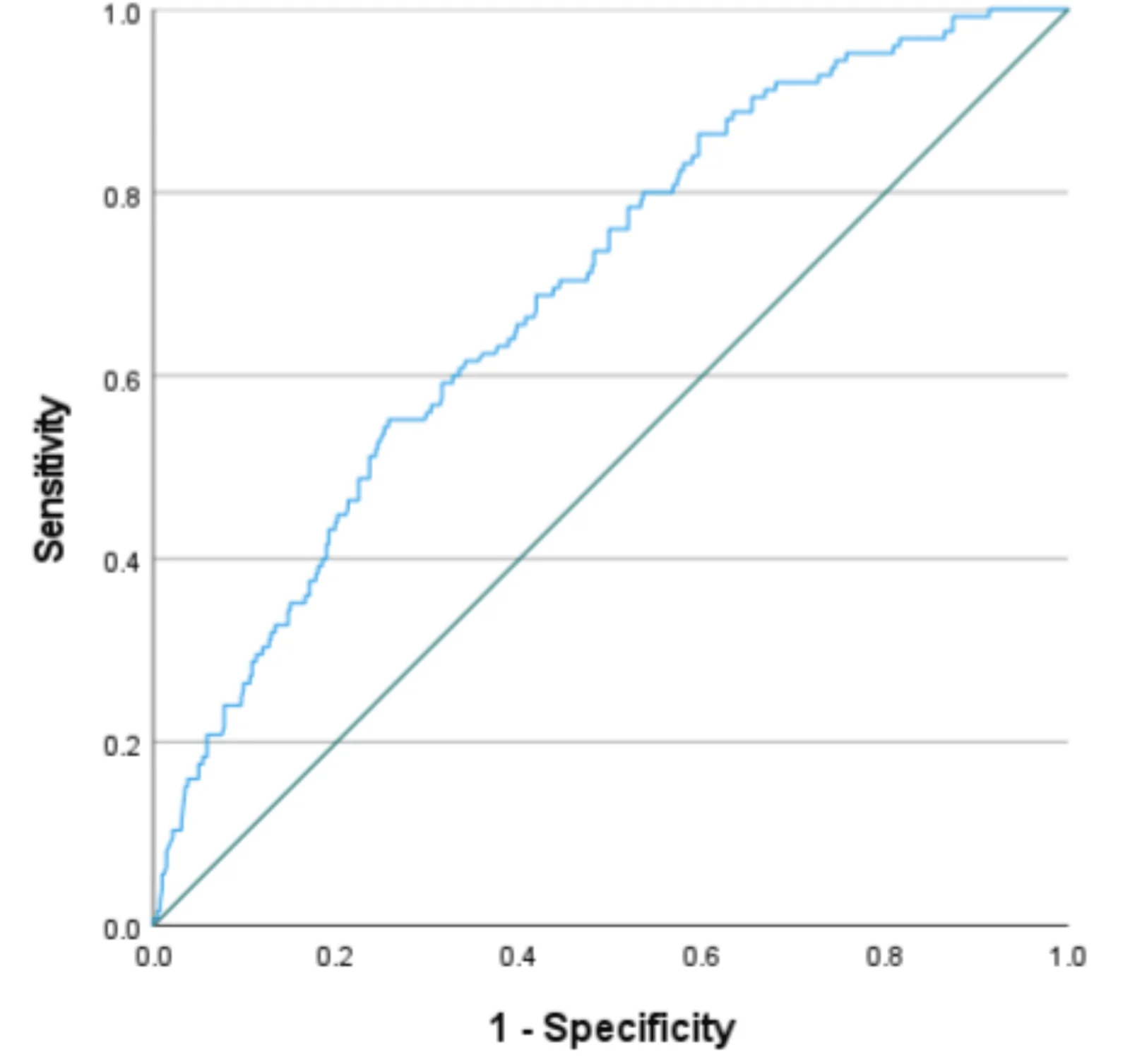
ROC Curve Analysis Analysis of TyG Index for Incident CVD Predication ROC: receiver operating characteristic; CVD: cardiovascular disease; TyG: triglyceride-glucose index

To predict incident CVD, ROC curve analysis was performed, as shown in Figure 1. The TyG index demonstrated moderate discriminative ability, with an AUC of 0.694 (95% CI: 0.643-0.744, p < 0.001). The optimal TyG cut-off value was 9.13, with a Youden index of 0.254. At this cut-off, the positive predictive value (PPV) was 31.5%, and the negative predictive value (NPV) was 86.3%.

Table 5 presents the results of a Cox proportional hazards regression model examining predictors of incident CVD (N = 553). The TyG index, which was a continuous variable in the unadjusted model, was strongly associated with an increased CVD risk (HR = 4.935, 95% CI (3.003-8.111), p < 0.001), a result that remained significant following adjustment for the covariates (adjusted HR = 4.597, 95% CI (2.695-7.841), p < 0.001). The relatively high hazard ratio may be attributable to clustering of metabolic risk factors and the hospital-based nature of the sample, which may have enriched the cohort for those at even greater risk. When analyzed categorically, participants in higher TyG quartiles had progressively greater risk compared to Q1 (reference), with adjusted hazard ratios of 3.170 (95% CI (1.532-6.557), p = 0.002) for Q2, 3.519 (95% CI (1.718-7.206), p = 0.001) for Q3, and 5.728 (95% CI (2.845-11.530), p < 0.001) for Q4, indicating a clear dose-response relationship. Hypertension was the only covariate that was statistically significant as a predictor of incident CVD (adjusted HR = 1.864, 95% CI (1.152-3.014), p = 0.011). Model fit improved after adjustment, with the -2 log-likelihood decreasing from 1,457.581 to 1,444.128 and the model χ² increasing from 39.952 (df = 3, p < 0.001) to 53.406 (df = 10, p < 0.001). In general, these results indicate that the TyG index emerged as a strong associated factor within this model (AUC = 0.694).

**Table 5:** Cox Proportional Hazards Regression Analysis for Incident CVD (N = 553) Q1 served as the reference category for all quartile comparisons. Time variable = months to CVD event or censoring (range: 12-29 months). Total events = 125 (22.6%); censored cases = 428 (77.4%). The adjusted model included age, gender, BMI, smoking status, hypertension, physical activity, and family history of CVD as covariates. Hypertension was the only covariate that was statistically significant in the adjusted model (HR = 1.864, p = 0.011). An AUC of 0.694 indicates moderate discriminative ability of the TyG index for predicting incident CVD. HR: hazard ratio; CI: confidence interval; TyG: triglyceride-glucose index; BMI: body mass index; AUC: area under the receiver operating characteristic curve; CVD: cardiovascular disease

Predictor/model component	Unadjusted HR	95% CI	p-value	Adjusted HR	95% CI	p-value
TyG index - continuous	4.935	3.003-8.111	<0.001	4.597	2.695-7.841	<0.001
TyG index - quartiles						
Q1 (reference)	1.000	-	-	1.000	-	-
Q2	3.072	1.492-6.324	0.002	3.170	1.532-6.557	0.002
Q3	3.668	1.808-7.442	<0.001	3.519	1.718-7.206	0.001
Q4	6.183	3.148-12.142	<0.001	5.728	2.845-11.530	<0.001
Covariates (adjusted model)						
Age (years)	-	-	-	1.016	0.997-1.036	0.092
Gender (female vs male)	-	-	-	0.814	0.571-1.160	0.254
BMI (kg/m²)	-	-	-	1.008	0.949-1.071	0.798
Smoking (yes vs no)	-	-	-	1.086	0.753-1.567	0.660
Hypertension (yes vs no)	-	-	-	1.864	1.152-3.014	0.011
Physical activity (active vs sedentary)	-	-	-	0.796	0.537-1.180	0.257
Family history (yes vs no)	-	-	-	0.828	0.563-1.218	0.339
Model fit statistics						
-2 log-likelihood	1,457.581	-	-	1,444.128	-	-
χ² (df)	39.952 (3)	-	<0.001	53.406 (10)	-	<0.001
Model discrimination						
Base model: Hypertension only	AUC = 0.636	95% CI: 0.578-0.694	-	-	-	-
Full model: TyG index + covariates	AUC = 0.694	95% CI: 0.638-0.750	-	ΔAUC vs hypertension-only: +0.058	-	-

## Discussion

The current study presents evidence that the TyG index may reflect an integrated metabolic signal and may provide additional information for cardiovascular risk assessment in non-diabetic adults. Although there is no overt hyperglycemia, participants with higher TyG values showed a significantly increased probability of developing cardiovascular events over time, highlighting that metabolic disruption is the antecedent to clinically defined disease states. This is consistent with large-scale prospective data, including the PURE study, which demonstrated that a higher TyG index was independently associated with an increased risk of cardiovascular events (adjusted HR 1.21, 95% CI 1.13-1.30) and incident diabetes in the general population. However, the stronger association observed in our study (adjusted HR 4.597) may reflect differences in study population, hospital-based recruitment, and the higher baseline cardiovascular risk profile of our cohort [14]. The study population was derived from a hospital-based cohort with a high prevalence of hypertension, suggesting that the findings may reflect a relatively higher-risk clinical population rather than a general community sample.

Among the most notable observations was the progressive increase in cardiovascular events in TyG quartiles. The results show a continuum, with even moderate levels of TyG being associated with a significant increase in risk. This is consistent with the concept of cardiometabolic degradation as a progressive process and its detection before typical diagnostic thresholds. An earlier study revealed that TyG is linearly related to the estimated 10-year atherosclerotic cardiovascular disease (ASCVD) risk, indicating that cardiovascular risk increases with TyG rather than at a particular cut-point. This is consistent with the concept of a continuum of cardiometabolic risk. These findings further support the use of the TyG index as a risk stratification marker rather than a simple binary indicator [17].

Notably, the association did not change after adjusting for demographic and clinical factors. This implies that the TyG index does not merely reflect the impact of known risk factors and may be considered a metabolic signal that combines insulin resistance, lipid abnormalities, and subtle glycemic dysregulation. Such an interpretation is consistent with earlier evidence demonstrating an independent association between TyG and cardiovascular outcomes, suggesting that it is a composite marker of cardiometabolic dysfunction [14,8]. In this context, the TyG index might be of value as an early metabolic stress marker, such as endothelial dysfunction, oxidative stress, and a pro-inflammatory state, which have been shown to result in vascular damage in previous studies [8,18].

Another interesting finding is that the traditional cardiovascular risk factors are attenuated in the multivariate model. The expected association between hypertension and cardiovascular events was observed, while other factors traditionally considered risk factors were generally independent and not significant. This finding corroborates earlier studies indicating that these factors operate together through common, interrelated processes and that mediation (e.g., after controlling for obesity) reduces their apparent independent effects [19,20]. This shift indicates that early metabolic markers, such as the TyG index, might detect risk at an earlier stage, though this is not as clinically salient as traditional markers. The heterogeneity can also stem from the use of a composite outcome, comprising hard and soft endpoints, which should be considered when interpreting the results.

Moderate discriminative performance found in the ROC analysis indicates that the TyG index cannot be considered an independent risk assessment tool but rather a complementary measure. This is primarily due to its simplicity, accessibility, and low cost, especially in an environment where other, more sophisticated biomarkers are not readily available. When used alongside other routine risk evaluation tools, this can increase the number of people identified as "low" or "intermediate" risk. Previous studies have shown that integrating the TyG index into current risk models significantly improves predictive accuracy, as evidenced by improvements in C-statistics and risk reclassification parameters. The results highlight its value as a complement, rather than a replacement, to traditional risk assessment tools [21].

The correlation analysis also helps interpret these results, as the TyG index is highly consistent with triglyceride levels and, on average, correlates with glucose and adiposity-related values. This provides biological plausibility for the TyG index as a surrogate marker of insulin resistance. This is supported by previous literature suggesting that the TyG index is a valid surrogate marker of insulin resistance, derived from TG and glucose levels, and correlates with several metabolic and CVDs [22,23]. Concurrently, its minimal association with BP indicates that it is a distinct metabolic pathway, not linked to hemodynamic action. This is corroborated by previous studies showing inconsistent results for the association between the TyG index and BP, with stronger associations for SBP than for DBP, especially in the obese group. The absence of correlation with DBP points to a metabolic, rather than hemodynamic, contribution of the TyG index, particularly the latter [24].

These findings, taken together, underscore the need to shift the focus to earlier stages of cardiometabolic dysfunction. The TyG index may serve as a practical and cost-effective screening tool for identifying individuals at increased cardiovascular risk before the onset of diabetes.

Limitations

Several limitations should be considered when interpreting the findings of this study. First, the use of non-probability convenience sampling in tertiary care hospitals may have introduced selection and referral bias, resulting in a study population with a higher baseline cardiometabolic risk profile than the general community and limiting the generalizability of the findings. Second, although no participants were lost to follow-up, the follow-up duration was relatively short and may not have captured longer-term cardiovascular outcomes. Third, other potentially important confounding factors were not adjusted for; therefore, complete correction for residual confounding was not possible. These included a detailed lipid profile (e.g., low-density lipoprotein cholesterol (LDL-C) and high-density lipoprotein cholesterol (HDL-C)), baseline medication use (e.g., statins and antihypertensive drugs), dietary habits, alcohol consumption, kidney function, and waist circumference or waist-to-hip ratio. Acute or chronic inflammatory conditions were not systematically assessed at baseline and may also have contributed to residual confounding. Furthermore, incident diabetes during follow-up was not systematically assessed; therefore, we could not determine whether progression to diabetes mediated the observed association between the TyG index and incident CVD. The primary outcome was a composite cardiovascular endpoint comprising myocardial infarction, stroke, angina, and documented coronary artery disease. Because event-specific analyses were not performed, the findings should be interpreted as reflecting overall cardiovascular risk rather than the risk of individual cardiovascular outcomes. In addition, the TyG index is a surrogate marker rather than a direct measure of insulin resistance, thereby precluding mechanistic interpretation, and the proportional hazards assumption was not formally evaluated using Schoenfeld residuals; therefore, the stability of the estimated hazard ratios should be interpreted with appropriate caution. A formal a priori sample-size calculation was not performed; however, the observed 125 incident events provided approximately 12.5 events per parameter in the adjusted Cox model. Finally, this study was conducted in Rawalpindi and Islamabad, which may limit the external validity of the findings to other populations.

Future directions

Future studies should validate these findings in larger, multicenter, and community-based populations to improve generalizability and external validity. Longer follow-up studies are needed to confirm the long-term prognostic value of the TyG index for cardiovascular events and mortality. Future research should also evaluate the incremental value of the TyG index when integrated with established cardiovascular risk prediction models and perform external validation in independent cohorts. In addition, advanced analytical approaches, including time-varying Cox regression and machine learning-based prediction models, may further refine cardiovascular risk stratification. Interventional studies are warranted to determine whether reducing the TyG index through lifestyle or pharmacological interventions translates into lower cardiovascular risk. Finally, incorporating detailed metabolic, inflammatory, and vascular biomarkers may provide further insight into the biological mechanisms linking the TyG index with CVD.

## Conclusions

The TyG index was independently associated with incident CVD and demonstrated moderate discriminatory ability in this hospital-based cohort of non-diabetic adults. Higher TyG levels were associated with progressively greater CVD risk across quartiles. These findings suggest that the TyG index may have potential as a complementary marker of cardiovascular risk; however, further validation in larger, community-based and ethnically diverse cohorts is required before clinical application can be recommended.

## Appendices

**Table 6:** Structured data collection proforma

Variable	Response
Participant ID	-
Date of Enrollment	-
Hospital/Center	-
Age (years)	-
Gender	-
Marital Status	-
Occupation	-
Socioeconomic Status	-
Weight (kg)	-
Height (m)	-
BMI (kg/m²)	-
Smoking Status	-
Hypertension	-
Blood Pressure (mmHg)	-
Physical Activity	-
Family History of CVD	-
Fasting Blood Glucose (mg/dL)	-
Fasting Triglycerides (mg/dL)	-
Known Diabetes	-
Known Cardiovascular Disease	-
TyG Index	-
Follow-up Date	-
Cardiovascular Event	-
Type of Event	-
Hospitalized	-
Confirmed by Medical Record	-
